# Cancer-Associated Thrombosis: A New Light on an Old Story

**DOI:** 10.3390/diseases9020034

**Published:** 2021-05-04

**Authors:** Sidrah Shah, Afroditi Karathanasi, Antonios Revythis, Evangelia Ioannidou, Stergios Boussios

**Affiliations:** 1 Department of Hematology/Medical Oncology, Medway NHS Foundation Trust, Windmill Road, Gillingham ME7 5NY, UK; sidrah.shah@nhs.net (S.S.); a.karathanasi@nhs.net (A.K.); antonios.revythis@nhs.net (A.R.); 2 Department of Paediatrics and Child Health, West Suffolk Hospital NHS Foundation Trust, Hardwick Lane, Bury St Edmunds IP33 2QZ, UK; e.ioannidou@nhs.net; 3 Faculty of Life Sciences & Medicine, School of Cancer & Pharmaceutical Sciences, King’s College London, London SE1 9RT, UK; 4 AELIA Organization, 9th Km Thessaloniki-Thermi, 57001 Thessaloniki, Greece

**Keywords:** cancer, thrombosis, thromboembolism, heparins, direct oral anticoagulant

## Abstract

Cancer-associated thrombosis (CAT) is a rising and significant phenomenon, becoming the second leading cause of death in cancer patients. Pathophysiology of CAT differs from thrombosis in the non-cancer population. There are additional risk factors for thrombosis specific to cancer including cancer type, histology, and treatment, such as chemotherapy. Recently developed scoring systems use these risk factors to stratify the degree of risk and encourage thromboprophylaxis in intermediate- to high-risk patients. Anticoagulation is safely used for prophylaxis and treatment of CAT. Both of these have largely been with low-molecular-weight heparin (LMWH), rather than the vitamin K antagonist (VKA); however, there has been increasing evidence for direct oral anticoagulant (DOAC) use. Consequently, international guidelines have also adapted to recommend the role of DOACs in CAT. Using DOACs is a turning point for CAT, but further research is warranted for their long-term risk profile. This review will discuss mechanisms, risk factors, prophylaxis and management of CAT, including both LMWH and DOACs. There will also be a comparison of current international guidelines and how they reflect the growing evidence base.

## 1. Introduction

The relationship between cancer and thrombosis was first reported by Bouilland in 1823 and Armand Trousseau later in 1865 [1,2]. Since then, the literature has further evaluated this significant relationship, naming it cancer-associated thrombosis (CAT), where cancer increases the risk of thrombosis [3]. Thrombotic events encompass venous thromboembolisms (VTEs), including deep vein thrombosis (DVT) and pulmonary embolism (PE), arterial thrombotic events (ATEs) and disseminated intravascular coagulation (DIC) [4]. Overall, cancer patients comprise 15–20% of patients who are diagnosed with VTE, and has steadily increased over the years [2,5]. Thrombosis has become the second leading cause of death in cancer patients, and conversely, cancer is a major cause of death in VTE patients [6,7,8,9]. The highest risk of VTE is in the initial months following cancer diagnosis and mortality is also highest one year after diagnosis [10]. The annual incidence of thrombosis in patients with cancer is higher at 0.5% compared to 0.1% in the general population [11]. Furthermore, cancer patients have a 21% annual risk of recurrent VTE and a 12% annual risk of bleeding complications, all of which can interrupt treatment options such as chemotherapy [12,13]. These clinical consequences emphasise the importance of effective prophylaxis and treatment of CAT for enhanced quality of life in cancer patients. This review will discuss pathophysiology and risk factors of CAT along with primary prophylaxis and management with comparison of current international clinical guidelines. 

## 2. Mechanisms of CAT

**Figure 1:**
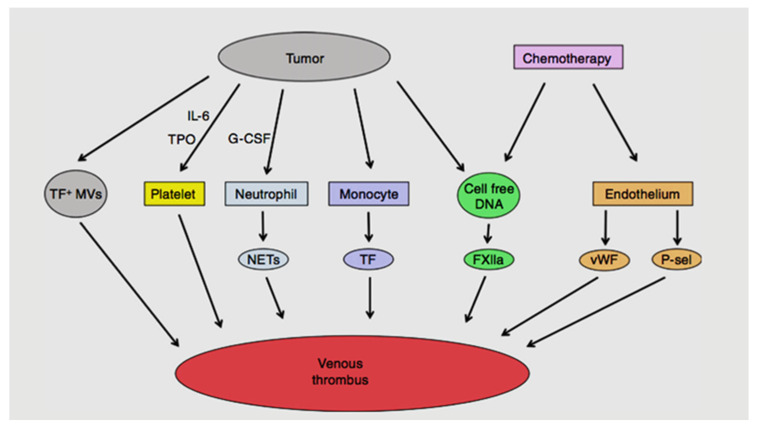
Mechanisms of cancer-associated thrombosis.

There are several direct and indirect mechanisms through which cancer promotes a hypercoagulable state and involves an interaction between cancer cells and the coagulation cascade [4,11]. Each of these components are discussed in further detail below and summarised in Figure 1. 

### 2.1. Coagulation Factors

Cancer cells can directly activate platelets through tumour-cell induced platelet aggregation (TCIPA) [3,14]. This involves secretion of thrombin, activation of coagulation factors V, VIII, XI and XIII and expression of adenosine diphosphate (ADP), which begins a cycle of further ADP release and subsequent activation and aggregation of platelets [3,15,16].

### 2.2. Tissue Factor

Along with platelets, tissue factor (TF) is a transmembrane protein that is significant for haemostasis [3]. In cancer, it is the primary initiator for the extrinsic pathway of the coagulation cascade and promotes tumour angiogenesis through production of VEGF [17]. It is expressed by malignant cells rather than normal vascular endothelium and can directly activate factor X—which produces thrombin and subsequent fibrin production—or factor VII when released by macrophages or monocytes [2,4,7,18]. Monocytes and macrophages express TF in an inactive form, which is then activated by agonists such as bacterial lipopolysaccharide and phorbol-12-myristate-13-acetate (PMA), and can activate the coagulation cascade as described above [19]. 

### 2.3. Microvesicles

Furthermore, TF can also be expressed on the surface of microvesicles. Microvesicles are membrane-enclosed vesicles that are released into the extracellular space by normal, apoptotic or malignant cells [4]. Tissue factor expressed on the surface of microvesicles is linked to the subsequent acceleration of coagulation and formation of thrombi [4,20]. Evidence of microvesicle release has predominantly been observed in pancreatic cancer patients, which could explain the higher incidence of CAT found in these patients [21]. 

### 2.4. Cancer Procoagulant 

Cancer procoagulant is a protease also expressed on the surface of tumour cells and directly activates factor X independently of factor VII [22]. It has been identified in breast cancer and leukaemia and thought to contribute to CAT, though further studies must be completed to quantify this association more thoroughly [4,23]. In addition, cancer causes an inflammatory response that leads to excess cytokine release [4,24]. The most prominent cytokines released are tumour necrosis factor alpha (TNF-a), interleukin-1 and interleukin-1b (IL-1b), and these induce the expression of von Willebrand factor and TF on vascular endothelial cells and monocytes [3,4,25,26,27]. Along with procoagulant activity, these cytokines can inhibit the thrombomodulin and protein C anticoagulation pathway [3,28]. Thrombomodulin binds to thrombin and causes a 20-fold faster inactivation of thrombin compared to free thrombin [29]. Furthermore, it activates protein C for the protein C pathway which inactivates coagulation factors V and VIII—two vital components of the coagulation cascade [30]. Through cytokines inhibiting these anticoagulant pathways, there is a greater risk of CAT [3,4]. 

### 2.5. Neutrophil Extracellular Traps

Neutrophil extracellular traps (NETs) are web-like structures released by neutrophils and composed of DNA fibres coated with histones and proteases [31]. Though they are a physiological response to infection, recent studies have identified their role in CAT. NETs can release von Willebrand factor through activation of endothelial cells, leading to platelet adhesion and aggregation essential for formation of thrombi [32,33]. NETs can also provide a direct platform and a scaffold for platelet adhesion and aggregation [34]. Cancers can create a systemic environment with release of cytokines causing a positive feedback loop that recruits more NETs [35]. Furthermore, NETs could be implicated in tumour progression, along with thrombosis, as their proteases may enhance tumour growth and further cytotoxic effects [31]. Mauracher et al. found that increasing levels of citrullinated histone H3 (H3Cit) were associated with higher incidences of VTE in the first six months and further strengthens the role of NETs in CAT [36].

## 3. Risk Factors

The risk factors for CAT can be broadly categorised into: patient characteristics, tumour related factors, treatment options and biomarkers. This is summarised in Table 1. This section will focus on risk factors for CAT and especially VTE as these are more commonly observed compared to ATEs.

### 3.1. Patient Characteristics

Similar to the general population, increasing age is a risk factor for VTE in cancer patients [4]. Retrospective cohort studies show patients aged 65 years had an increased risk of VTE compared to younger patients [37,38]. Other risk factors include female sex, multiple co-morbidities such as heart failure, renal disease and infection—infection, especially, is found to be one of the strongest risk factors for VTE [38]. Khorana et al. also studied VTE rates across different ethnicities and found patients of black ethnicity had the highest rates of VTE at 5.1% [38]. Furthermore, after one episode of VTE, the annual risk of subsequent VTE was also highest in the black population at 36.7% compared to 26.8% for other ethnicities (*p* = 0.07) [38]. This was also seen in another retrospective study where black patients had an incidence of 1.8% of VTE compared to 0.6% in the white patients [39]. Further studies are needed to investigate this link. However, this may be due to higher levels of factor VIII, von Willebrand factor and D-dimer along with increased thrombophilias (especially sickle cell disease) in this patient population [40]. Other patient factors increasing risk of VTE are immobility—causing stasis of venous blood flow—a prior history of VTE and a BMI ≥ 35 kg/m^2^ [41].

### 3.2. Tumour-Related Factors

There are various tumour-specific factors that increase VTE risk in cancer patients. These include the site of the cancer, its staging and histology. Higher incidences of VTE are found in cancers of the pancreas, stomach, kidney, ovary, lung, uterus and brain [42]. Furthermore, haematological malignancies are now also showing higher incidences of VTE [29]. Even among these cancers, the rates vary significantly. The California Cancer Registry found VTE rates were 20% and 17% in patients with advanced pancreas and stomach cancer respectively, compared to 2.8% and 0.9% in advanced breast and prostate cancers respectively [43]. With VTE rates differing with site of cancer, this suggests there are tumour specific influences on CAT. As the cancer advances and metastasises, the prothrombotic tendency and the risk of VTE are higher, likely due to a bulkier tumour load obstructing venous flow [3,44]. This is also suggested by a population-based study, which found the risk of VTE was 58-fold in cancer patients with distant metastases compared to non-cancer patients—this is significantly higher than the four-fold increased risk of VTE in cancer patients without metastases [45].

The tumour type can impact the site of the VTE. For example, a literature review of VTE in colon cancer by Otani et al. found cancers of the ascending and transverse colons had VTE in the superior mesenteric vein compared to widely diffuse colon cancer developing VTE in the portal vein [46,47]. Histology of certain cancers also affects VTE rates. Chew et al. compared VTE rates in non-small cell lung cancer and found 9.9% of patients with adenocarcinoma developed VTE six months following diagnosis compared to 7.7% of patients with squamous cell carcinoma [48]. It is unclear why certain histological types of different cancers can increase of VTE. However, in ovarian cancer the clear cell histology increases hypercoagulability, whereas the epithelial ovarian cancer increases VTE risk by increasing TF expression [49,50].

### 3.3. Cancer Treatment

The highest risk of VTE is within the first three to six months following diagnosis and this may be due to many types of treatment being initiated soon after diagnosis [4,29]. Central venous catheters (CVCs) are useful to take blood samples from and provide chemotherapy or other intravenous treatment. However, they can cause venous stasis or injure endothelium on entry, and therefore lead to catheter-related thrombosis with the rate of symptomatic DVT between 0.3% and 28% [4,51]. Surgery and hospitalisation also increase the risk of VTE through immobility leading to venous stasis and several processes contributing to a prothrombotic state [3]. Agnelli et al. found that surgery increased the risk of DVT 2-fold and fatal PE 3-fold when compared to non-cancer patients [52]. A total of 40% of VTE occurred after 21 days of surgery and overall death rate was 1.72%—death was caused by VTE in 46.3% of the patients. Risk factors for these post-operative VTEs included: age over 60 years, previous episode of VTE, advanced malignancy, immobility over three days and anaesthesia lasting over two hours [34]. The risk of postoperative VTE has been reduced with increasing compliance to thromboprophylaxis, and this could explain some reports finding surgery to be less of a risk factor for VTE compared to other factors [53,54]. Higher incidences of VTE were found in abdominal, pelvic and lower limb orthopaedic surgeries [4,55].

Chemotherapy is a significant risk factor for CAT and may have contributed to its increasing incidence due to mechanisms that directly harm the vascular epithelium, reduce anticoagulant substances or increase the procoagulant protein [4,56,57,58]. Chemotherapy increases the risk of CAT by six to seven-fold and examples are both cytotoxic and targeted chemotherapy [3,39]. Cisplatin doubled the risk when used in combination chemotherapy for gastroesophageal cancer compared to other combinations using another platinum-based drug oxaliplatin [59]. Cisplatin is reported to induce endothelial apoptosis and platelet activation, and up-regulate prothrombotic factors [60]. Other chemotherapy agents increasing thrombosis risk include thalidomide, lenalidomide, tamoxifen, L- asparaginase and 5-Fluorouracil [3,4]. This is seen in myeloma patients where treatment with thalidomide or lenalidomide combined with steroids can lead to a VTE risk of 8% to 27% [61]. Radiotherapy is also used with or without chemotherapy for treatment of cancer and can increase VTE risk [3]. Guy et al. found that of their cohort of cancer patients, 13% had received radiotherapy prior to developing VTE [62]. Radiotherapy has been found to cause a pro-coagulant response and haemostasis through increasing D-dimer, activated factor VIII, TF and von Willebrand factor, the latter of which can also cause endothelial dysfunction and thrombosis [63,64].

### 3.4. Hormonal and Molecular Risk Factors

Oestrogen and thyroid hormones can increase the risk of CAT [3]. Thyroid hormone T4 activates the platelet surface integrin avb3, which promotes platelet aggregation and degranulation [65]. Therefore, T4 has been identified as a factor that allows tumour cells to proliferate and metastasise [53]. This could explain the link between thyroid hormones and CAT through enhanced platelet activation [3]. Oestrogen is also implicated in VTE as demonstrated by hormone replacement therapy (HRT) and the combined oral contraceptive pill (COC) causing an increased risk of VTE [3,66,67,68]. Higher VTE rates have been seen in endometrial and breast cancers where oestrogen levels are also higher [3]. Platelets and megakaryocytes express oestrogen receptors and studies completed on mice show that higher doses of oestrogen can lead to platelet activation [69,70]. Other mechanisms discussed include high doses of oestrogen increasing coagulation factors VII and X by 170% of baseline, increasing fibrinogen by 10–20% and reducing protein S by 50% [71].

Oncogenes can also increase CAT risk [3]. Oncogenic mutations of the K-RAS and p53 tumour suppressor genes increase TF expression through release of TF containing microvesicles, and also increase procoagulant and proangiogenic activity [72]. Furthermore, a retrospective study by Ades et al. found that mutations activating K-RAS were associated with a three-fold increase of DVT incidence [73]. Mutated oncogenes and oncoproteins, such as Human Papillomavirus (HPV) E6, can also upregulate the vascular endothelial growth factor (VEGF), leading to hyperpermeable tumour blood vessels that create an environment to stimulate the coagulation cascade [74,75].

### 3.5. Biochemical Markers

Several biomarkers have been identified to predict the risk of VTE. Higher pre-chemotherapy platelet count ≥350 × 10^9^/L and leucocyte count ≥ 11 × 10^9^/L along with haemoglobin levels < 100 g/L have been associated with higher risk of VTE [76]. Elevated platelets could be observed in both venous and arterial thromboembolism as they are the main cell type in thrombosis and may represent a higher pro-inflammatory state in cancer patients [77]. Another explanation is that cancer cells release abnormal mucin glycoproteins—these mucins can use *p*-selectin, adhesion molecules expressed by activated platelets, as templates to aggregate activated platelets [78]. These mucins can also activate L-selectin molecules—they activate leucocytes, and the resulting activated leucocytes can activate platelets through an unknown mechanism, thus starting the coagulation cascade [78]. Leucocytes may also bind with activated platelets to form microthrombi within the circulation; these microthrombi may then adhere to the endothelium and create a foci where further thrombi can form and adhere to [79]. These mechanisms are possible hypotheses to explain the role of these biomarkers.

Elevated D-dimer is also predictive of higher VTE; a prospective cohort study found colorectal cancer patients with elevated D-dimer of >0.3 mg/L had a 20% incidence of DVT in one year compared to 5% for patients without [80]. Elevated D-dimer represents increased haemostasis, fibrin deposition and degradation and consequently is a significant prognostic biomarker [81]. Another significant biomarker is TF as many cancers express an abnormally high level of this molecule [82]. Expression of TF is induced by inflammatory cytokines rather than usual vascular cells and this initiates coagulation via the extrinsic pathway [11,83]. A small retrospective study found resected pancreatic tumour specimens had high TF expression and a VTE rate of 26.3% compared to 4.5% in patients with low TF expression [84]. Furthermore, TF expression is also associated with increased expression of VEGF and therefore angiogenesis for tumour cells [7,62]. Other biomarkers correlated with higher risk of VTE include: a higher C-reactive protein (CRP), soluble *p*-selectin and pro-thrombin fragment 1.2 [11,22].

## 4. Primary Prophylaxis of CAT

### 4.1. Scoring Systems

Based on the risk factors listed above, Khorana et al. derived a risk stratifying score to predict VTE risk in cancer patients [76]. This scoring system was developed from a prospective observational study using 2701 ambulatory patients receiving chemotherapy, and then validated in an independent cohort of 1365 patients from the same study [76]. It has been validated in other studies, including the Vienna CATS, which incorporated additional factors D-dimer and *p*-Selectin [41,85]. The PROTECHT (PROphylaxis of ThromboEmbolism during CHemoTherapy) score also adds to the Khorana score to include platinum or gemcitabine-based chemotherapy to account for high VTE rates in patients receiving these [85,86]. This is demonstrated in Table 2. The Khorana score identifies a high-risk score as ≥ 3; however, studies have found limitations to using this cut-off with low sensitivity in lung and pancreatic cancer [87,88]. Furthermore, 50% of patients usually fall into the intermediate risk score and physicians struggle to treat these patients as opposed to those who score as low-risk or high-risk [89]. The Vienna CATS and PROTECHT score aim to improve the predictive value and studies confirm that they have an improved ability to identify higher risk of VTE [89,90]. Another prospective cohort study compared these scores and found there was poor discriminatory prediction of VTE when compared to objectively confirmed VTE over a six-month period; however, the Vienna CATS and PROTECHT scores appeared to distinguish between low- and high-risk patients better due to incorporation of biomarkers and chemotherapy use [85]. Though there are conflicting results of these scoring systems, calculating the risk of thrombosis in individual patients would allow significant benefit from thromboprophylaxis.

### 4.2. Low-Molecular-Weight Heparin

Multiple studies discuss prophylaxis for thrombosis in cancer and these are summarised in Table 3. The double-blind, placebo-controlled PROTECHT study evaluated the efficacy of nadroparin, a low-molecular-weight heparin (LMWH), as prophylaxis for CAT in ambulatory patients receiving chemotherapy. A total of 1150 patients were randomised in a 2:1 ratio to receive daily subcutaneous nadroparin 3800 IU or placebo for either the entire duration of chemotherapy or four months [91]. Of the patients in the placebo group, 3.9% had CAT compared to 2% of the patients receiving nadroparin (*p* = 0.02) [91]. Higher thrombotic events occurred in lung and pancreatic cancers compared to breast, ovarian, gastrointestinal and head and neck [7,91]. However, major bleeding was observed in 0.7% of patients receiving nadroparin versus none in the placebo group [91]. This highlights the difficult trade-off between the benefits of preventing CAT and increased risk of bleeding with thromboprophylaxis in cancer patients [92]. Another double-blind, multi-centre trial (SAVE-ONCO) focussed on efficacy of the LMWH semuloparin in patients with metastatic or locally advanced cancer receiving chemotherapy [93]. 3212 patients were randomly assigned to placebo or to receive 20 mg once daily with a median treatment duration of 3.5 months; VTE was observed in 20 of 1608 patients (1.2%) receiving semuloparin versus the 55 of 1604 patients (3.4%) receiving placebo (*p* < 0.001) [93]. In contrast to the PROTECHT study, major bleeding rates were similar with 19 of 1589 patients (1.2%) in the semuloparin group and 18 of 1583 patients (1.1%) in the placebo group [93]. Here, the authors concluded semuloparin reduces the risk of thrombosis in patients receiving chemotherapy without significant increases in major bleeding risk [93]. A meta-analysis by Di Nisio et al. concluded that while semuloparin is not commercially available, primary thromboprophylaxis with LMWH reduced incidence of VTE in ambulatory patients receiving chemotherapy [94]. However, although results are encouraging, more studies need to focus on the risk-to-benefit ratio of LMWH due to its implications of major bleeding before routine prophylaxis can be rolled out [6,94].

### 4.3. Direct Oral Anticoagulants

With direct oral anticoagulants (DOACs) increasingly used for treatment of VTE and PE, studies have assessed their role in prophylaxis of CAT. Levine et al. conducted a pilot study of whether apixaban was associated with any adverse outcomes in 125 patients receiving chemotherapy; they concluded that 93.5% of the 93 patients receiving apixaban did not have major or clinically relevant non-major bleeding (CRNMB) [95]. Therefore, they showed apixaban was a safe option for thromboprophylaxis in cancer patients. The Apixaban for the Prevention of Venous Thromboembolism in High-Risk Ambulatory Cancer Patients (AVERT) trial also assessed efficacy of apixaban in cancer patients [96]. In this randomised, double-blind clinical trial, the Khorana score was used to identify 563 cancer patients with intermediate-to-high risk for VTE (score ≥ 2). Of these, 288 patients received apixaban 2.5 mg twice a day for 180 days and 275 patients were in the placebo group. VTE occurred in 12 patients (4.2%) in the treatment group compared to 28 patients (10.2%) in the placebo group (*p* < 0.001) [96]. Furthermore, major bleeding occurred in six patients (2.1%) receiving apixaban compared to three patients (1.1%) in the placebo group during the treatment period [96]. Higher rates of haematuria and gastrointestinal and gynaecological bleeding were observed with apixaban and major bleeding was mainly in patients with gastrointestinal or gynaecological cancer [96]. Like prophylaxis with LMWH, use of apixaban may be associated with lower incidence of VTE but does not address the challenge of major bleeding risk in cancer patients.

### 4.4. Thromboprophylaxis in Surgical Patients

There are conflicting studies for the use of thromboprophylaxis in cancer patients. In contrast, the Enoxaparin and Cancer (ENOXACAN) I and II trials evaluated prophylaxis in patients undergoing abdominal or pelvic surgery [97,98]. The ENOXACAN I trial found 14.7% of patients receiving enoxaparin once daily developed VTE compared to 18.2% of patients receiving unfractionated heparin three times a day [97]. In the double-blind ENOXACAN II trial, 332 patients receiving abdominal or pelvic surgery were given 40 mg enoxaparin daily for one week and then randomly assigned to receive either enoxaparin or placebo for the remaining three weeks. Of the 167 patients receiving enoxaparin for one week (placebo group), 20 (12.0%) patients developed VTE compared to eight of the 165 (4.8%) patients receiving enoxaparin for four weeks [98]. At three months follow-up, VTE had occurred in 23 (13.8%) of the 167 placebo patients and nine (5.5%) of the 165 patients receiving enoxaparin. Relative risk reduction remained 60% (95% CI 10–82% in the first month and 95% CI 17–81% in third month) and no major bleeding complications were observed. With these trials, prophylactic LMWH is widely used for at least thirty days in cancer patients undergoing major surgery [6].

## 5. Current Management of CAT

Patients with CAT are a growing population that require anticoagulation treatment and this treatment differs to that of non-cancer patients. Until the early 2000s, oral vitamin K antagonists (VKA) were primarily used for CAT treatment [6]. Studies have since focused on the use of LMWH and DOACs for CAT as drug interactions, renal and hepatic dysfunction and thrombocytopenia were significant implications of VKA use [6]. LMWH inhibits the final common pathway of the coagulation cascade through activation of antithrombin III, which then inhibits factors Xa and IIa [99]. Several studies have assessed the use of LMWH in CAT with the CANTHANOX trial being one of the first [100]. This randomised, multi-centre trial compared recurrent VTE and major bleeding events between 146 patients receiving warfarin or the LMWH enoxaparin. Patients were either given 1.5 mg/kg of enoxaparin once daily or warfarin 6 mg to 10 mg orally to maintain an international normalised ratio (INR) between 2.0 and 3.0 for three months. 15 (21.1%) patients of the warfarin group experienced major bleeding and recurrent VTE compared to seven (10.5%) patients receiving enoxaparin (95% CI 4.3–20.3%, *p* = 0.09) [100]. Furthermore, six patients died due to haemorrhage in the warfarin group compared to none receiving enoxaparin. The study concluded that a full dose of enoxaparin is just as effective as but may be safer than warfarin for long-term treatment of thrombosis in cancer patients.

The CLOT (Randomised Comparison of Low-Molecular-Weight Heparin versus Oral Anticoagulant Therapy for the Prevention of Recurrent Venous Thromboembolism in Patients with Cancer) trial has defined LMWH as treatment of CAT since 2003 [101]. It is a large randomised clinical trial comparing VKA to a LMWH in 672 patients from 48 centres in eight countries [6,102]. 336 patients received dalteparin 200 IU/kg once daily for one month, followed by 150 IU/kg once daily for five months. 336 patients assigned to the VKA group initially received dalteparin 200 IU/kg once daily for five to seven days followed by VKA for six months. 27 (9%) patients treated with dalteparin had recurrent VTE events compared to 53 (17%) in the VKA group (*p* = 0.002). VTE occurred in 20 patients receiving warfarin when the INR was below 2.0. The Kaplan–Meier estimate of the probability of recurrent VTE at six months was 9% in patients receiving dalteparin and 17% in the VKA group. Furthermore, there was no significant difference in major bleeding with 6% in the dalteparin group and 4% in the VKA group (*p* = 0.27). The CLOT trial highlighted treatment with VKA can be challenging due to the requirement of maintaining a therapeutic INR. The INR was in range for only 46% of the time, lower than the recommended time of >60% [6,101]. Consequently, this led to LMWH being first-line therapy for CAT and still widely used.

The CATCH (Comparison of Acute Treatments in Cancer Haemostasis) study added to the CLOT trial further and became the largest trial to compare LMWH with VKA [103]. 900 cancer patients receiving treatment or with a history of treatment in the last six months were randomised into receiving tinzaparin 175 IU/kg once daily or warfarin adjusted to INR for six months [103]. In contrast to the CATCH study, the difference in recurrent VTE rates was not statistically significant with 31 (7.2%) patients receiving tinzaparin and 45 (10.5%) patients on warfarin (*p* = 0.07). Major bleeding was found in 12 (2.7%) patients of the tinzaparin group and 11 (2.4%) in the warfarin group (*p* = 0.77). However, CRNMB was statistically significant with 49 (10.9%) patients in the tinzaparin group compared to 69 (15.3%) in the warfarin group (*p* = 0.004). Differences between the CLOT and CATCH trial may be attributed to fewer thrombotic episodes than expected in the CATCH trial, which would reduce the potential for benefit of LMWH [103]. Less VTE may be due to the patient characteristics in the CATCH trial: less metastatic disease, previous history of thrombosis and use of chemotherapy [6,103]. Nevertheless, the results of both trials pointed to similar ideas that LMWH could be a safe and effective treatment for CAT [6]. This is highlighted by time spent in the therapeutic range of INR being 46% in the CATCH trial; again, this emphasises the difficulty of achieving optimal therapeutics for anticoagulation with VKA.

Several studies support monotherapy of LMWH for CAT, but the DALTECAN study highlights the challenges of long-term LMWH treatment [104]. This observational study evaluated the safety of dalteparin of more than six months in cancer patients with a definitive diagnosis of DVT or PE. They were given subcutaneous dalteparin 200 IU/kg once daily for the first month, then prefilled syringes according to weight for the remaining 11 months. Major bleeding occurred in 34 (10.2%) of 334 patients, with incidence higher in the first six months at 1.7% compared to 0.7% in months 7–12. The highest incidence was in the first month at 3.6%. Bleeding occurred mostly in the brain and gastrointestinal and genitourinary tracts. Furthermore, recurrence of thrombosis occurred in 37 (11.1%) patients over 12 months—29 (8.7%) patients experienced this in the first six months compared to eight (4.1%) in months 7–12. Again, the incidence per patient-month was highest in the first month at 5.7%. The primary cause of mortality was underlying cancer (105 patients) followed by recurrent PE (four patients) and bleeding (two patients). Though the study was limited with no control group, it illustrated the persistent risk of recurrent thrombosis despite LMWH anticoagulation and high risk of major bleeding. Though the majority of guidelines support LMWH treatment for CAT, these complications are significant for increasing both patient morbidity and mortality.

The use of DOACs for VTE has been a significant achievement, but there are limited studies on its use in CAT [11]. They can directly inhibit thrombin (dabigatran etexilate) or factor Xa (apixaban, rivaroxaban, betrixaban) [3]. Their benefits over LMWH include frequent laboratory monitoring of INR not required, oral administration instead of subcutaneous and fewer food and drug interactions [3,11]. Randomised clinical trials have recently compared DOACs to LMWH in the treatment of CAT. The Hokusai VTE Cancer trial compared edoxaban, an oral factor Xa inhibitor, to subcutaneous dalteparin for treatment of VTE in cancer patients for up to 12 months [105]. In this randomised controlled trial, 1046 patients with DVT or PE were given either LMWH for five days followed by oral edoxaban 60 mg once daily or subcutaneous dalteparin 200 IU/kg once daily for one month followed by 150 IU/kg once daily for the remainder of the time. The study found edoxaban was noninferior to dalteparin for recurrent VTE or major bleeding. 67 (12.8%) of the 522 patients receiving edoxaban had a primary-outcome event (VTE or major bleeding) compared to 71 (13.5%) of the 524 patients receiving dalteparin (*p* = 0.006 for noninferiority, *p* = 0.87 for superiority). Recurrent VTE was identified in 41 (7.9%) patients in the edoxaban group and 59 (11.3%) patients in the dalteparin group. In contrast, major bleeding was higher in patients receiving edoxaban at 36 (6.9%), compared to 21 (4.0%) in the dalteparin group. This was due to higher rates of upper gastrointestinal bleeding in patients with gastrointestinal cancer receiving edoxaban (*p* = 0.02).

Therefore, while DOACs are as effective as LMWH for the treatment of CAT, higher bleeding risk especially in gastrointestinal cancers needs to be considered. The SELECT-D (Anticoagulation Therapy in Selected Cancer Patients at Risk of Recurrence of Venous Thromboembolism) trial also highlighted these concerns [106,107]. Here, 406 patients with DVT or PE were randomly assigned to receive either rivaroxaban 15 mg twice daily for three weeks then 20 mg once daily or dalteparin 200 IU/kg daily for one month then 150 IU/kg for a total of six months. Again, recurrent VTE rates were lower in the rivaroxaban group with eight patients compared to 18 in the dalteparin group. The cumulative recurrent VTE rate at six months was 4% in patients receiving rivaroxaban and 11% in those receiving dalteparin. However, major bleeding was higher in the rivaroxaban group with a cumulative six-month incidence of 6% compared to 4% in the dalteparin group. This was also observed to be the highest in the upper gastrointestinal sites and in patients with gastrointestinal cancers. Furthermore, there was a three-fold increase in CRNMB with rivaroxaban and these required medical intervention or disrupted activities of daily living [107]. As in the Hokusai VTE Cancer trial, a DOAC has been identified as an effective alternative to LMWH [6,107].

Agnelli et al. conducted the multinational, randomised Caravaggio trial comparing oral apixaban with subcutaneous dalteparin in 2020 [108]. Patients with DVT or PE received either 10 mg apixaban twice daily for the first seven days followed by 5 mg twice daily or dalteparin 200 IU/kg once daily for the first month and then 150 IU/kg once daily over a six-month period. The primary outcome was objectively confirmed recurrent VTE, and this occurred in 32 of 576 (5.6%) patients receiving apixaban compared to 46 of 579 (7.9%) patients in the dalteparin group (*p* < 0.001 for noninferiority). Major bleeding occurred in 22 patients (3.8%) of the apixaban group and in 23 patients (4.0%) receiving dalteparin (*p* = 0.60). This study concluded that apixaban was noninferior to dalteparin for treatment of recurrent VTE. Furthermore, in contrast to previous studies, major bleeding was similar for both groups including gastrointestinal bleeding. This is particularly interesting given that one third of the cancers in this study were of gastrointestinal origin. These results could be encouraging as it is more convenient for patients to take DOACs orally compared to subcutaneous LMWH and this could increase adherence to anticoagulation treatment [109]. However, given there is conflicting evidence on the risk of major bleeding with DOACs, each patient must be assessed individually-especially if they have gastrointestinal cancer. Guidelines have been updated to account for DOACs in treatment of CAT and these are discussed below.

## 6. Comparison of Current Clinical Guidelines

Historically, LMWH has been the treatment of choice for CAT, but this is changing with the addition of DOACs into many guidelines. The international guidelines discussed are from the American Society of Clinical Oncology (ASCO), the National Comprehensive Cancer Network (NCCN), the International Society of Thrombosis and Haemostasis (ISTH), the European Society of Medical Oncology (ESMO) and the National Institute for Health and Care Excellence (NICE). The majority of these guidelines have now shifted away from VKA first line and incorporate the use of DOACs for CAT; they are summarised in Table 4 [110,111,112,113,114,115,116,117]. The ESMO guidelines are the only guidelines from this list that do not support the use of DOACs in the treatment of CAT—this may be because they were published in 2011 before the recent release of evidence base on DOACs [113]. DOACs included as first-line and equal to LMWH range from apixaban, edoxaban and rivaroxaban. However, guidelines caution for their use in gastrointestinal cancers due to the higher risk of bleeding; this is corroborated with the findings of the SELECT-D and Hokusai VTE Cancer trials [105,107]. Furthermore, they are to be used only if there are no interactions, no risk of bleeding and patients have a creatinine clearance of over 30 mL/min consistent with the DOAC clinical trials [118]. In general, treatment is advised for at least three months and can be continued up to six months; all guidelines advise to assess duration on an individual case-by-case basis. Trials have generally focussed on six-month treatment outcomes of DOACs and consequently data is inconclusive beyond twelve months [118]. Further research on longer duration of treatment is therefore needed to bridge this gap of information.

Each guideline, excluding the NICE guideline, sections prophylaxis into different patient groups. These are prophylaxis for hospitalised medical patients, surgical patients and ambulatory patients receiving chemotherapy. The NICE guideline is limited because it is not specific for patients with cancer-it includes general VTE prophylaxis for all patients except specific advice on pancreatic cancer and myeloma patients [116,117]. In the other guidelines, thromboprophylaxis in cancer is generally LMWH or UFH for hospitalised patients with acute medical illness or surgical patients as per older studies and guidelines [118]. Thromboprophylaxis for ambulatory cancer patients does not have clear-cut or assertive wording, but DOACs apixaban and rivaroxaban are being recommended in high-risk ambulatory patients receiving chemotherapy [110,111,112,114,115]. In future, DOACs may become even more prominent in further CAT guidelines due to their convenience and cost-effectiveness over LMWH. In the Netherlands, the six-month cost of rivaroxaban and dalteparin preventing VTE recurrence was compared [119]. The study concluded rivaroxaban could be saving more than $11 million Euros primarily due to savings on treatment costs with lower VTE events from rivaroxaban use. Furthermore, Li et al. found that low dose rivaroxaban or apixaban, when compared to placebo, were cost-effective prophylaxis for six months in intermediate-to-high risk patients, especially with a Khorana score ≥ 3 [120]. Again, this was due to lower cost from VTE events and complications. Though DOACs are showing promise, their safety profile with respect to major bleeding risk still needs additional evaluation in future studies before they overtake LMWH [118].

## 7. Conclusions

There is a well-defined relationship between cancer and thrombosis. The rising incidence of CAT is concerning as it is the second leading cause of death in cancer patients. CAT has a different pathophysiology compared to thrombosis in the non-cancer population. There are complex interactions between platelets, host cells, tumour cells, factors of the coagulation cascade, proteins such as TF and many more. Furthermore, there are multiple risk factors for CAT which can be divided into patient characteristics, tumour related factors, treatment options and biomarkers. Identification of these risk factors has allowed development of risk scores to quantify the risk of CAT and whether thromboprophylaxis is justified. International guidelines now advise prophylaxis in certain subgroups of cancer patients. These include hospitalised acutely unwell medical patients, surgical patients and intermediate- to high-risk ambulatory patients receiving chemotherapy. LMWH is still the mainstay for prophylaxis in hospitalised medical and surgical patients, but DOACs are being considered in prophylaxis for ambulatory patients receiving chemotherapy. Furthermore, LMWH became the preferred choice of treatment of CAT over VKA for many years, but the guidelines are now incorporating the use of DOACs. DOACs are more convenient than LMWH and may become the preferred treatment choice for patients. Large trials have confirmed their efficacy in preventing recurrence of thrombosis; however, the concerns over their bleeding risk need to be studied further. There is a fine balance between successful anticoagulation and bleeding risk in cancer patients. Consequently, further data on the long-term risk-benefit profile of the DOACs for CAT will be monumental for future clinical practice and reduce morbidity and mortality in this patient population.

## Figures and Tables

**Table 1:** Risk factors for cancer-associated thrombosis.

Patient Characteristics	Tumour-Related Factors	Treatment Factors	Hormonal and Molecular Factors	Biomarkers
Increasing age	Site of tumour	Surgery	Thyroid hormones	Platelet count ≥ 350 × 10^9^/L
Female sex	Tumour staging	Hospitalisation	Oestrogen	Leucocyte count ≥ 11 × 10^9^/L
Black ethnicity	Tumour histology	Chemotherapy	Oncogenes—K-RAS, p53	Elevated D-dimer
Comorbidities-heart failure, renal disease and infection		Radiotherapy	Oncoproteins—HPV E6	High expression of TF from cancer cells
Immobility		Central venous catheters		Elevated CRP
Previous VTE				Soluble *p*-selectin
BMI ≥ 35 kg/m^2^				Prothrombin fragment 1.2

Abbreviations: VTE: venous thromboembolism, HPV: human papillomavirus, TF: tissue factor, CRP: C-reactive protein, BMI: body mass index.

**Table 2:** Predictive risk scores for venous thromboembolism in cancer patients.

Patient Characteristics	Khorana Score	Vienna CATS Score	PROTECHT Score
Very high-risk cancer (pancreas, stomach)	2	2	2
High risk cancer (lung, gynaecological, lymphoma, bladder, testicular)	1	1	1
Haemoglobin level < 10 g/dL or use of red cell growth factors	1	1	1
Pre-chemotherapy platelet count ≥ 350 × 10^9^/L	1	1	1
Pre-chemotherapy leucocyte count ≥ 11 × 10^9^/L	1	1	1
BMI ≥ 35 kg/m^2^	1	1	1
D-dimer > 1.44 mg/L	-	1	-
Soluble *p*-selectin > 53.1 ng/L	-	1	-
Gemcitabine chemotherapy	-	-	1
Platinum-based chemotherapy	-	-	1

Khorana score: high risk ≥ 3 points, intermediate 1–2 points, low risk 0 points. Abbreviations: CATS: cancer and thrombosis study, PROTECHT: Prophylaxis of Thromboembolism during Chemotherapy, BMI: body mass index.

**Table 3:** Summary of studies in primary prophylaxis of cancer-associated thrombosis.

Trial	Year	Number of Patients	Intervention	VTE Rates	Major Bleeding Events
PROTECHT [91]	2008	1150	Nadroparin 3800 IU once daily versus placebo	Nadroparin: 2.1% Placebo: 3.9% (*p* = 0.02)	Nadroparin: 0.7% Placebo: 0%
SAVE-ONCO [93]	2012	3212	Semuloparin 20 mg once daily versus placebo	Semuloparin: 1.2% Placebo: 3.4% (*p* < 0.001)	Semuloparin: 1.2% Placebo: 1.1%
Levine et al. [95]	2012	125	Apixaban once daily * versus placebo	Apixaban: 0% Placebo: 10.3%	Apixaban: 2.2% Placebo: 3.4%
AVERT [96]	2019	563	Apixaban 2.5 mg twice daily for 180 days versus placebo	Apixaban: 4.2% Placebo: 10.2% (*p* < 0.001)	Apixaban: 2.1% Placebo: 1.1%
ENOXACAN II [98]	2002	332	Enoxaparin 40 mg daily for 31 days versus placebo (enoxaparin only for first 10 days)	Enoxaparin: 4.8% Placebo: 12.0% (*p* = 0.02)	Enoxaparin: 0.4% Placebo: 0%

* 32 patients to 5 mg, 29 patients to 10 mg, 32 patients to 20 mg; Abbreviations: VTE: venous thromboembolism, PROTECHT: Prophylaxis of Thromboembolism during Chemotherapy, AVERT: Apixaban for the Prevention of Venous Thromboembolism in High-Risk Ambulatory Cancer Patients, ENOXACAN: Enoxaparin and Cancer.

**Table 4:** Current treatment guidelines for cancer-associated thrombosis.

Guidelines/ Reference/Year	Recommendations			
	Prophylaxis for hospitalised cancer patients	Prophylaxis for surgical cancer patients	Prophylaxis for ambulatory patients receiving chemotherapy	Treatment of thrombosis in cancer patients
ASCO/ [110]/2020	Pharmacological prophylaxis should be offered for patients with acute medical illness in the absence of contraindications and bleeding. Without additional risk factors, prophylaxis may be offered if no contraindications or bleeding. Should not be offered for minor procedures, chemotherapy infusions, patients having stem cell/bone marrow transplants.	All patients undergoing major surgery should be offered UFH or LMWH preoperatively if no contraindications or bleeding. Prophylaxis should be at least 7–10 days and up to 4 weeks if major abdominal or pelvic surgery in high risk patients.	Routine pharmacological prophylaxis should not be offered to all outpatients. High-risk outpatients with a Khorana score of ≥2 may be offered: apixaban, rivaroxaban or LMWH if no contraindications or bleeding. Patients receiving thalidomide or lenalidomide for multiple myeloma should be offered aspirin or LMWH if low-risk or LMWH for high-risk patients.	Initial treatment with UFH, LMWH, rivaroxaban or fondaparinux. LMWH preferred over UFH if CrCl ≥ 30 mL/min. Long-term anticoagulation with LMWH, edoxaban or rivaroxaban for at least 6 months. VKA may be used if above contraindicated. Treatment beyond 6 months for patients with metastatic cancer or receiving chemotherapy and should be assessed individually. DOACs should not be offered in GI or GU malignancy.
NCCN/ [111,112]/2020	Recommend LMWH, UFH or fondaparinux with or without PCD for all hospitalised patients if no contraindications or bleeding. If pharmacological prophylaxis contraindicated, use PCD.	Prophylaxis with LMWH, fondaparinux or UFH (category 1) is recommended. Consider preoperative UFH or LMWH for abdominal or pelvic surgery for up to 4 weeks	No routine prophylaxis if low risk. Consider apixaban or rivaroxaban for high-risk patients with a Khorana score ≥ 2 for up to 6 months. Myeloma patients receiving iMiDs should be offered aspirin if low risk (SAVED < 2 points) or LMWH or VKA for high risk (SAVED ≥ 2 points)	Apixaban, edoxaban with initial LMWH for 5 days, rivaroxaban if no GI malignancy. LMWH (dalteparin, enoxaparin) for GI malignancy or UFH if CrCl < 30 mL/min. Dabigatran with LMWH for initial 5 days if above contraindicated. Fondaparinux or VKA may also be used. Treatment for at least 3 months or as long as active cancer/cancer treatment
ESMO/ [113]/2011	Recommend UFH, LMWH or fondaparinux for patients with acute medical illness and confined to their bed.	Prophylaxis is recommended in major surgery with LMWH or UFH. LMWH should be given for one month after major abdominal or pelvic surgery. Mechanical prophylaxis may be added and should only be used as monotherapy if LMWH or UFH are contraindicated.	Routine prophylaxis for advanced cancer or patients receiving adjuvant chemotherapy is not recommended but may be considered in high-risk ambulatory patients. Consider LMWH, aspirin or warfarin (target INR 1.5) for patients receiving thalidomide with dexamethasone or chemotherapy for multiple myeloma.	Initial treatment LMWH 200 IU/kg once daily or UFH. If CrCl < 30 mL/min, then either UFH or LMWH with anti-Xa monitoring. Long term anticoagulation with LMWH for 6 months is considered safe and more effective than VKA.
ISTH/ [114,115]/2019	Recommend UFH, LMWH and fondaparinux for all patients with acute medical illness. LMWH is preferred over UFH due to lower major bleeding risk. Consider fondaparinux if previous history of HIT. DOACs are not recommended for prophylaxis. Prophylaxis should not be offered for minor procedures or chemotherapy infusions.	Recommend LMWH if CrCl ≥ 30 mL/min or UFH. Start 2–12 h preoperatively and continue for 7–10 days. Extend prophylaxis to 4 weeks if undergoing major laparotomy or laparoscopic surgery and there is high VTE risk with low risk of bleeding.	Suggest apixaban or rivaroxaban if Khorana score ≥ 2 and no bleeding risk e.g., GI cancer. Use for up to 6 months after beginning chemotherapy. If apixaban or rivaroxaban contraindicated or if high bleeding risk, suggest LMWH instead.	Initial treatment with LMWH if CrCl ≥ 30 mL/min, UFH or fondaparinux. Rivaroxaban or edoxaban (after 5 days of LMWH/UFH) is also recommended if CrCl ≥ 30 mL/min and no risk of GI or GU bleeding. Long-term anticoagulation with LMWH or DOAC for at least 6 months. DOACs should be cautioned in GI malignancies.
NICE/ [116,117]/2018, 2020	No specific guideline for cancer patients. General guidelines are written for all patients unless specified. Offer prophylaxis for a minimum of 7 days to acutely ill patients. Offer LMWH as first line and if contraindicated, use fondaparinux instead.	No specific guideline for cancer patients in particular. General guidelines are written for surgical patients.	Do not offer to ambulatory patients receiving chemotherapy unless increased VTE risk from a factor other than cancer. Consider LMWH as VTE prophylaxis in patients receiving chemotherapy for pancreatic cancer. Consider aspirin or LMWH for myeloma patients receiving thalidomide, lenalidomide or pomalidomide with steroids.	Consider a DOAC for confirmed VTE in active cancer. If DOAC unsuitable, consider LMWH or LMWH with VKA until INR is 2.0 on 2 consecutive readings, then VKA alone. Offer for 3–6 months and then review according to need if longer treatment needed.

Abbreviations: ASCO: American Society of Clinical Oncology, UFH: unfractionated heparin, LMWH: low molecular weight heparin, CrCl: creatinine clearance, VKA: vitamin K antagonist, DOAC: direct oral anticoagulant, GI: gastrointestinal, GU: genitourinary, NCCN: National Comprehensive Cancer Network, PCD: pneumatic compression device, ESMO: European Society of Medical Oncology, INR: international normalised ratio, ISTH: International Society of Thrombosis and Haemostasis, HIT: heparin-induced thrombocytopenia, VTE: venous thromboembolism, NICE: National Institute for Health and Care Excellence.
